# Serum levels of fetuin-A are negatively associated with log transformation levels of thyroid-stimulating hormone in patients with hyperthyroidism or euthyroidism: An observational study at a medical center in Taiwan: Erratum

**DOI:** 10.1097/MD.0000000000015298

**Published:** 2019-04-12

In the article, “Serum levels of fetuin-A are negatively associated with log transformation levels of thyroid-stimulating hormone in patients with hyperthyroidism or euthyroidism: An observational study at a medical center in Taiwan”,^[1]^ which appears in Volume 97, Issue 46 of *Medicine*, the unit of serum fetuin-A levels should be μg/mL, instead of ng/mL.
